# Preventing mental health conditions in adolescents living with HIV: an urgent need for evidence

**DOI:** 10.1002/jia2.25556

**Published:** 2020-08-31

**Authors:** Christina A Laurenzi, Sarah Skeen, Sarah Gordon, Olamide Akin‐Olugbade, Nina Abrahams, Melissa Bradshaw, Amanda Brand, Stefani du Toit, G J Melendez‐Torres, Mark Tomlinson, Chiara Servili, Tarun Dua, David A Ross

**Affiliations:** ^1^ Institute for Life Course Health Research Department of Global Health Stellenbosch University Cape Town South Africa; ^2^ Peninsula Technology Assessment Group College of Medicine and Health University of Exeter Exeter United Kingdom; ^3^ School of Nursing and Midwifery Queens University Belfast United Kingdom; ^4^ Department of Mental Health World Health Organization Geneva Switzerland; ^5^ Department of Maternal, Newborn, Child and Adolescent Health and Ageing World Health Organization Geneva Switzerland

**Keywords:** adolescents, interventions, public health, social support, mental health, psychosocial interventions

## Abstract

**Introduction:**

As adolescents transition from childhood to adulthood, they experience major physical, social and psychological changes, and are at heightened risk for developing mental health conditions and engaging in health‐related risk behaviours. For adolescents living with HIV (ALHIV), these risks may be even more pronounced. Research shows that this population may face additional mental health challenges related to the biological impact of the disease and its treatment, the psychosocial burdens of living with HIV and HIV‐related social and environmental stressors.

**Discussion:**

Psychosocial interventions delivered to adolescents can promote positive mental health, prevent mental health problems and strengthen young people’s capacity to navigate challenges and protect themselves from risk. It is likely that these interventions can also benefit at‐risk populations, such as ALHIV, yet there is little research on this. There is an urgent need for more research evaluating the effects of interventions designed to improve the mental health of ALHIV. We highlight four priorities moving forward. These include: generating more evidence about preventive mental health interventions for ALHIV; including mental health outcomes in research on psychosocial interventions for ALHIV; conducting intervention research that is sensitive to differences among ALHIV populations and involving adolescents in intervention design and testing.

**Conclusions:**

More robust research on promotive and preventive mental health interventions is needed for ALHIV. Programmes should be informed by adolescent priorities and preferences and responsive to the specific needs of these groups.

## 1 INTRODUCTION

As adolescents transition from childhood to adulthood, they undergo major physical, social and psychological changes [1]. Physical changes, which include puberty and rapid brain development, take place in the context of newly developing autonomy, responsibility and decision‐making abilities. This transition is also influenced by a complex set of socio‐economic factors, including family and cultural environments, which interact with each other and shape adolescents’ health trajectories and vulnerabilities [2]. During this dynamic yet precarious life stage, adolescents are at heightened risk for developing mental health conditions (such as depression and anxiety) and engaging in health‐related risk behaviours. As many as 10% to 20% of people will develop mental health conditions during adolescence, and it is estimated that up to 50% of all mental health conditions start before the age of 14 [3]. Self‐harm, which includes suicidal behaviours, is among the top three causes of death for 15‐ to 19‐year‐old boys and girls globally [4]. Furthermore, mental health conditions during this period are associated with a range of risk behaviours, including tobacco and alcohol use, drug misuse, risky sexual behaviours and violence [5, 6], the effects of which may persist throughout the life course.

Adolescents living with HIV (ALHIV) are at an even greater risk of developing mental health conditions and risk behaviours [7]. Worldwide, an estimated two million adolescents are living with HIV, with over 80% of them residing in sub‐Saharan Africa [8]. Depression, anxiety, hopelessness and fear for the future are common in this population, which makes mental health a vital area of concern for ALHIV [9]. Research shows that these risks are manifold, related to the biological impact of the disease and its treatment, the psychosocial burden of living with HIV and HIV‐related social and environmental stressors. From a biological perspective, for adolescents who acquired HIV perinatally, the effects of the virus on brain development persist into adolescence [10], and there is mixed evidence on whether highly active antiretroviral therapy can slow or reverse damage to the developing brain [11, 12]. ALHIV also face numerous psychosocial challenges. Many young people with perinatally acquired HIV first learn they are living with HIV during adolescence, which can be highly stressful and create familial tensions if they blame their parents for their condition [13]. Relatedly, ALHIV may also experience grief from losing one or both parents, or other caregivers, contributing to their own expectations and fears of illness and death [9, 14]. Social and environmental stressors include experiencing heightened stigma and isolation; adolescents may also be increasingly required to manage their own treatment adherence [15, 16]. ALHIV engaging in romantic and sexual relationships for the first time need to grapple with how to disclose their status to partners and protect against potential fears of rejection [17]. Additionally, adolescents living in vulnerable households with others who are also living with HIV may have additional mental health needs that intersect with experiences of poverty and illness [18].

There is additional evidence that the mental health of ALHIV affects other domains of their health and wellbeing. In general, clinical outcomes for adolescents tend to be worse than those of adults, and adolescents have poorer levels of adherence to antiretroviral therapy (ART) and thus higher viral loads [19, 20]. Evidence from adult populations reveals a complex relationship between mental health and HIV, including poor physiological and psychological outcomes related to factors such as disease progression, medication side effects, social isolation and the financial burden of being ill [21]. The same mechanisms that can contribute to poor health in adults living with HIV are likely to affect ALHIV; however, improving mental health can also foster better HIV outcomes such as adherence and retention in care [22], especially for adolescents [23].

## 2 DISCUSSION

Adolescence is thus a critical time to intervene with this vulnerable group – to prevent mental conditions, to promote positive mental health and to strengthen young people’s capacity to navigate challenges and protect themselves from risk. Psychosocial interventions have been identified as beneficial when delivered to universal, or general, adolescent populations: these interventions adopt a psychological, behavioural, and/or social approach to improve psychosocial wellbeing and reduce the risk of poor mental health outcomes [24]. Our meta‐analysis found that psychosocial interventions that included specific components (emotional regulation, interpersonal skills, mindfulness, assertiveness training, problem solving, stress management, and alcohol and drug education) were associated with more successful programme outcomes for adolescent mental health [24].

However, there is less research about the impact of these types of interventions among targeted groups, such as ALHIV, who are likely to have specific, additional psychosocial support needs. From an equity perspective, it is critical to consider if and how psychosocial interventions might benefit special populations, including ALHIV. The same skills taught and practiced in a psychosocial intervention for a universal population of adolescents – for example, navigating changing peer dynamics or setting goals – may take on new significance as they help ALHIV disclose their status to a trusted peer, or conceptualize a healthy, fulfilling adult life. With a growing number of adolescents globally – including the largest number of children born with HIV to survive into adolescence – this imperative is even greater.

*Helping Adolescents Thrive* (HAT), a joint initiative between the World Health Organization and UNICEF, represents one such attempt to provide more evidence for both universal and targeted interventions for adolescent mental health. A 2019 evidence review linked with HAT, conducted in preparation for the development of the WHO Guidelines on Mental Health Promotive and Preventive Interventions for Adolescents, found only three randomized controlled trials targeting mental health outcomes for ALHIV ages 10 to 19 [25, 26, 27], shown in Table 1. As the burden of HIV and mental health continues to persist among this population, there is an urgent need for research evaluating the effects of interventions designed to improve the mental health of ALHIV. Drawing primarily on this review, we have distilled four recommendations to guide future research in this area.

**Table 1:** Summary of studies included in review

Author and year	Article name	Country	Programme Intent	Total sample (N), % girls	Age (mean, sd)	Study population description	Mental health outcomes measured^a^	Summary of findings as reported by authors
Bhana *et al*. (2014)	The VUKA family programme: piloting a family‐based psychosocial intervention to promote health and mental health among HIV infected early adolescents in South Africa	South Africa	RCT to prevent depression and anxiety; promote communication and mental wellbeing	65, 49.2%	11.57, n/s	Recruited children between 10 and 14 years old enrolled in HIV care at the hospital and aware of their HIV status at two clinical sites in KwaZulu‐Natal	Positive mental health (mental wellbeing and mental functioning) Mental disorders (depression and anxiety)	At 3 months post‐intervention, intervention participants showed a significant improvement in positive mental health (youth/caregiver communication comfort, β = 0.796, *p* = 0.002 and communication frequency, β = 0.478, *p* = 0.09). Mental disorders showed a non‐significant reduction in symptoms (depression, β = 0.736, *p* = 0.417).
Webb *et al*. (2018)	Mindfulness instruction for HIV‐infected youth: A randomized controlled trial	United States	RCT to prevent stress, aggression and lower CD4 count; promote mindfulness, mental functioning, life satisfaction and adherence	72, 45.8%	18.71, 2.31	Adolescent participants were eligible if they received their medical care at one of the clinics, did not have any significant cognitive, behavioural, or psychiatric disorders and had a current CD4 count above 200	Mental disorders (depression and anxiety) Positive mental health (mental wellbeing and mental functioning) Adherence to antiretroviral treatment Aggressive, disruptive and oppositional behaviours	At three months post‐intervention, intervention participants showed significant improvements in positive mental health (mindfulness, β = 0.65, 95%CI [0.06,1.24], *p* = 0.03, problem‐solving coping β = 0.49, 95%CI [0.05, 0.92], *p* = 0.03, and life satisfaction, β = 0.57, 95%CI [0.01, 1.13], *p* = 0.05) and aggressive, disruptive and oppositional behaviours (aggression, β=−0.89, 95%CI [−1.41, to 0.37], *p* = 0.002).
Willis *et al*. (2019)	Effectiveness of community adolescent treatment supporters (CATS) interventions in improving linkage and retention in care, adherence to ART and psychosocial wellbeing: a randomized trial among adolescents living with HIV in rural Zimbabwe	Zimbabwe	RCT to promote adherence, self‐esteem and quality of life	94, 59.6%	10 to 15, n/s	Adolescents living with HIV, receiving ART at three selected clinic sites	Positive mental health (mental wellbeing) Adherence to antiretroviral treatment	At 12‐month follow‐up, intervention participants reported significant increases in positive mental health (confidence, self‐esteem and self‐worth, point difference = 0.49, 95%CI [0.313,0.667], *p* < 0.001) and adherence to ART (OR = 3.934, 95%CI [1.404, 11.02], *p* = 0.0087). Significant increases in quality of life were reported for both intervention participants (point difference = 0.29, 95%CI [0.031, 0.549], *p* = 0.028) and control participants (point difference = 0.26, 95%CI [0.061, 0.459], *p* = 0.011).

^a^ These measures are worded accordingly to the outcome specifications in the review.

### 2.1 Invest in high‐quality research to test the effectiveness of interventions to prevent mental health conditions and promote positive mental health for ALHIV

There is a clear need to invest in more research about the mental health of ALHIV. Increased HIV‐related research on adolescents regarding new strategies for biomedical treatment and adherence, given their unique risk profile and susceptibility to worse HIV outcomes, is promising [28]. However, there are glaring omissions in the evidence on mental health for ALHIV. Mental health, as a critical foundation for overall wellbeing and quality of life, must be prioritized in research and interventions with ALHIV. We argue that there should be an equally robust approach to generating evidence about how best to promote positive mental health, and prevent mental conditions and risk behaviours, in this population. Integrating services that consider and address mental health into existing HIV services that adolescents routinely access is one way to bridge this gap. Recent reviews have identified the need for integrating mental health services into HIV care in high‐burden settings [29], especially for adolescents [7]. Integrated models, which might consist of multidisciplinary teams coordinating care in a “one‐stop shop”, or service providers managing two‐way referrals between HIV and mental health care, have been found to be both feasible and acceptable in high‐burden, low‐resource settings [30, 31].

There is also a need to build process data into studies evaluating effectiveness, to give stakeholders and funders a multidimensional understanding of the complexity of programming with ALHIV. Process measures might include attendance, dosage and coverage of sessions; delivery characteristics; delivery and participation costs; content relevance; contextual barriers and enablers; implementer competence; and implementer soft skills. For adolescents who are more difficult to reach, more innovative engagement methods may be necessary. These include adolescents who do not access clinical care or HIV treatment consistently, those in age‐disparate relationships, those living in vulnerable family circumstances and those who are involved in sex work or transactional relationships [32, 33]. Research using process data holds important lessons for understanding why certain interventions may be easier to implement in given populations, or why some interventions may show limited evidence of effectiveness.

### 2.2 Include mental health outcomes in studies of the effectiveness of psychosocial interventions to promote HIV treatment adherence and reduce risk behaviours

There is a large body of evidence relating to behavioural and psychosocial interventions for ALHIV; however, these studies rarely report on mental health outcomes specifically, often focusing on treatment adherence and sexual and reproductive health outcomes [34, 35, 36, 37]. Existing interventions tend to be specifically designed to promote adherence to ART and prevent risky sexual behaviours such as unprotected intercourse [38], which are seen as essential to supporting adolescent health and preventing onward transmission. At the same time, these interventions tend to employ content and delivery mechanisms that are also likely to benefit mental health, such as decision‐making skills, self‐esteem, coping skills, support networking, psychoeducation and peer support [35, 39, 40].

As such, it is critical that measures that capture self‐reported or parent‐reported mental health are included in these types of studies as primary or secondary outcomes. In the absence of these measures and accompanying data, it is impossible to know whether psychosocial interventions have positive, null, or potentially negative effects on participants’ mental health. Similarly, the effectiveness of adherence and risk behaviour interventions may be mitigated by underlying mental health outcomes that are not being accurately considered or incorporated into analysis: for example, the impact of self‐harm or suicidal ideation on non‐adherence. Embedded within this recommendation is a note of caution about context. As psychosocial interventions for ALHIV are increasingly implemented in sub‐Saharan African settings, selecting the appropriate mental health measures and ensuring their validity among the research population is essential to gathering high‐quality data [41].

### 2.3 Conduct intervention research that is sensitive to individual differences and specific needs among heterogenous populations of ALHIV

While many ALHIV share a common set of vulnerabilities, acknowledging the diversity and complexity of this group is critical when considering how to design and implement programmes. Differences in mode of infection, age group (younger versus older) and gender, as well as additional adolescent comorbidities, may affect how adolescents engage with an intervention. Evidence shows that as children born with HIV transition into adolescence, the way that they relate to their HIV status and engage in treatment behaviours may change, as they gain autonomy, come to terms with their illness and take control of their own health care‐seeking [42, 43]. Adolescents who acquire HIV later in their teens may experience a different set of challenges that complicate their ability to initiate care, with underlying mental health problems contributing to poorer health and adherence outcomes [44]. Depending on mode of infection – and on the duration of their illness, access to social support networks and other intersecting life stressors and risk behaviours – ALHIV may have ways of relating to their illness that are diverse. Research that is attuned to differences by mode of HIV infection could provide a more nuanced approach to improving mental health and could identify means of engaging and retaining adolescents in these interventions.

### 2.4 Involve and empower adolescents in intervention development and testing

Actively involving and engaging adolescents throughout the conceptualization and implementation stages of interventions is important for ensuring interventions are acceptable and relevant – and ultimately effective. Special considerations should be made to develop adolescent‐friendly interventions that actively include adolescents at all stages, and not to retrofit interventions used with adult populations. Co‐production strategies, such as adolescent advisory boards, allow adolescents to drive how content is delivered and what messages are emphasized [45, 46]. As this field develops, adolescents should take a lead role in crafting interventions that speak to their distinct needs and are also informed by cutting‐edge evidence.

## 3 CONCLUSIONS

ALHIV are faced with many potential risks to their mental health, yet there are few evaluations of promotive and preventive mental health interventions for this group. This group is a critical population to engage further through more frequent, robust research that can inform the development of new interventions. We call for more high‐quality research into interventions for ALHIV that is informed by adolescent priorities and preferences and responsive to the specific needs of this group.

## COMPETING INTERESTS

The authors declare no competing interests.

## AUTHORS’ CONTRIBUTIONS

CAL conceptualized and drafted the manuscript, and coordinated further writing and editing among the co‐authors. SS led the team that conducted the systematic reviews in collaboration with the WHO. SG, OAO, NA, MB, AB, SD and GJMT, along with CAL, all worked on the systematic review team and made important contributions to identifying eligible articles, extracting data, assessing risk of bias, analysing data, and conducting literature reviews to contextualize findings. MT, CS, TD and DAR provided leadership and input to the review team throughout the duration of the project and supported in conceptualizing the manuscript. All authors reviewed the manuscript and provided feedback at various stages, and read and approved the final manuscript.

## ABBREVIATIONS

ALHIV, adolescents living with HIV; ART, antiretroviral therapy; HIV, human immunodeficiency virus; WHO, World Health Organization.
